# Age-period-cohort analysis of smoking prevalence trends among career military personnel in South Korea

**DOI:** 10.18332/tid/196477

**Published:** 2024-12-07

**Authors:** Sunju Jung, Heewon Kang, Sung-il Cho

**Affiliations:** 1 Department of Public Health Science, Graduate School of Public Health, Seoul National University, Seoul, Republic of Korea; 2 Institute of Health and Environment, Graduate School of Public Health, Seoul National University, Seoul, Republic of Korea

**Keywords:** military personnel, age-period-cohort analysis, smoking prevalence, intrinsic estimator, South Korea

## Abstract

**INTRODUCTION:**

This study evaluated smoking trends among career military personnel in South Korea. After a tobacco tax increase in 2015, the tobacco control program for career military personnel expanded significantly. This study explores long-term smoking trends among career military personnel through age-period-cohort (APC) analysis within the context of targeted tobacco control policies.

**METHODS:**

This secondary data analysis utilized data from 12052 individuals aged 19––54 years, identified as career military personnel in the Korea Community Health Survey (KCHS) from 2008 to 2022. APC analysis with the intrinsic estimator (IE) method was conducted to address multicollinearity and accurately assess the contributions of age, period, and birth cohort to smoking prevalence.

**RESULTS:**

Smoking prevalence significantly decreased among career military personnel, from 46.2% in 2008 to 34.1% in 2022. The highest prevalence of smoking was initially observed in the age group of 22–24 years. Although overall prevalence declined, a slight increase was observed in the age group of mid-30s to early 40s, suggesting that smoking behavior varied across age groups. A significant decrease occurred between 2014 and 2016, with individuals born in the 1970s exhibiting the highest smoking prevalence, and those born after 1980 demonstrating a notably lower smoking prevalence.

**CONCLUSIONS:**

Although smoking prevalence has declined, the distinct age, period, and cohort characteristics of career military personnel necessitate tailored tobacco control policies that consider the various aspects of military life.

## INTRODUCTION

The health of military personnel is crucial for national security, operational readiness, and training program effectiveness^1^. In the military context, smoking is a prevalent health behavior linked to diminished operational capacity and significant socioeconomic losses, underscoring the need for effective smoking cessation policies^2^.

The military of South Korea comprises conscripted soldiers and career military personnel. The latter includes commissioned officers, warrant officers, and noncommissioned officers who serve voluntarily. In the military, tobacco control policies primarily target conscripted soldiers.

Over the past decade, the demographic composition of active-duty forces has significantly shifted. The proportion of conscripted soldiers has decreased, whereas that of career military personnel has increased by approximately 12%, from 27.6% in 2012 to 39.5% in 2022^3^. This shift is important because career military personnel, who experience prolonged exposure to the military environment, encounter unique stressors such as work-related stress, family separation, and pressure to conform to military culture^1,4^. These factors frequently lead them to utilize tobacco as a coping mechanism and to facilitate their integration into the military community^4^.

The pivotal role of career military personnel in the military hierarchy and their potential to influence smoking culture, enable them to enhance the effectiveness of military smoking cessation policies. While tobacco control policies in the military have been shown to reduce smoking prevalence among personnel, the risk of relapse after the cessation of such policies remains a concern^5^. Therefore, it is crucial to methodically examine the effects of tobacco control policies on smoking prevalence among South Korea’s career military personnel.

Although various aspects of military personnel’s smoking behaviors have been investigated, the focus has predominantly been on conscripted soldiers, emphasizing short-term smoking habits in specific units or small groups^6-9^ and during particular periods^4,8,10,11^. In contrast, smoking behaviors among career military personnel remain understudied, despite their potential long-term influence on smoking culture within the military due to their extended service and leadership roles. Moreover, military service itself significantly influences the rate of smoking among men^12,13^; indeed, men who have served in the South Korean military exhibit a higher smoking prevalence than their civilian counterparts^14,15^.

In response to the increasing awareness of smoking’s negative effects, the South Korean military has transitioned away from a tobacco-permissive culture^14^ by adopting tobacco control policies^16^. These policies include the complete abolition of duty-free tobacco allocations in the military in 2009 and a tenfold increase in the budget for smoking cessation programs in 2015. The timelines of these policies are provided in Supplementary file Table 1. These changes have resulted in a steady decrease in smoking prevalence among military personnel.

The demographic and cultural factors described above underscore the importance of investigating long-term smoking trends among career military personnel. Accordingly, we conducted an APC analysis using the IE method to manage multicollinearity among age, period, and birth cohort variables. Our aim was to provide insight into the unique smoking behaviors of career military personnel, thereby contributing to the development of targeted smoking cessation strategies.

## METHODS

### Data source and study population

This study utilized secondary data from the KCHS, an annual cross-sectional survey managed by the Korea Disease Control and Prevention Agency (KDCA). The analysis period spanned 15 years, from 2008 to 2022. Among adults aged 19–54 years, a total of 12052 individuals who identified their occupation as career military personnel were selected as participants. As the weighting of the KCHS is designed to ensure regional representativeness, it was not applied in this study, which specifically aimed to estimate values for the military population. The KCHS dataset used in this study was approved by the IRB of the KDCA and the study was exempted from review by the Seoul National University in South Korea.

### Measures

We employed a model to analyze smoking trends among career military personnel, focusing on variables such as age, period, and cohort, based on responses to occupation classification and smoking status. Until 2018, smoking status was defined by the question: ‘Do you currently smoke?’ and thereafter as ‘Do you currently smoke conventional cigarettes?’. Individuals who answered ‘smoke daily’ or ‘smoke occasionally’ were categorized as ‘current smokers’, those who answered ‘having smoked in the past but not currently’ as ‘former smokers’, and those who answered ‘never smoked’ as ‘never smokers’. To further refine the classification, an ‘ever smoker’ category was introduced, encompassing individuals classified as ‘current’ and ‘former’ smokers, thereby providing a comprehensive view of lifetime smoking history. The age range of 19–54 years, was divided into twelve 3-year groups, and the study period (2008–2022) was segmented into five 3-year intervals. Birth cohorts, calculated by subtracting the individual’s age from the survey year, were grouped into sixteen 3-year intervals.

### Statistical analysis

APC analysis uses a log-linear Poisson regression model to estimate smoking prevalence (λ_i_), incorporating age, period, and cohort effects as:

*log (λ_i_) = Intercept + β_1_×Age_i_ + β_2_×Period_i_ + β_3_×Cohort_i_*

where λ_i_ is the smoking prevalence for observation i, and the coefficients β_1_, β_2_, and β_3_ represent the effects of age, period, and cohort, respectively. Prevalence ratios were obtained by exponentiating the estimated coefficients, offering a relative measure of smoking prevalence across different age, period, and cohort groups. This approach enables comparisons that reveal specific trends within each category. This model encounters multicollinearity due to the relationship among age, period, and cohort variables (cohort = period - age)^17,18^. To address this issue, the IE method was utilized based on its accuracy and management of multicollinearity in APC analysis, facilitating exploratory analysis and interpretation of complex variable interactions based on the principles of principal component analysis^18-20^. In line with this approach, equal 3-year intervals were applied to age, period, and cohort variables to enable clear comparisons across groups and ensure consistency throughout the analysis. Additionally, the IE method has been used to study smoking prevalence among young adults^21^ and adolescents^22,23^, and for comparison between genders^24^.

Goodness-of-fit statistics were computed to select the optimal model for analyzing smoking prevalence among career military personnel (Table 1). The age + period + cohort (IE) model proved optimal for analyzing smoking prevalence among career military personnel, based on its lowest AIC and deviance/df values for optimal fit.

Statistical analysis was primarily conducted using R software, version 4.3.2, for data processing, analysis, and visualization. Additionally, the *apc_ie* module in STATA SE, version 18 (StataCorp LP, College Station, TX, USA), was employed for age-period-cohort analysis.

**Table 1:** Goodness-of-fit statistics for age-period-cohort models for smoking prevalence among career military personnel

*Model*	*Deviance*	*df*	*AIC*	*Deviance/df*
Age (years)	119.243	48	503.417	2.484
Age + period	52.859	44	445.032	1.201
Age + cohort	24.855	33	439.029	0.753
Age + period + cohort (intrinsic estimator)	20.613	30	7.346	0.687

df: degrees of freedom. AIC: Akaike information criterion. Deviance/df: model fit per degree of freedom, with lower values indicating better fit.

## RESULTS

**Figure 1:**
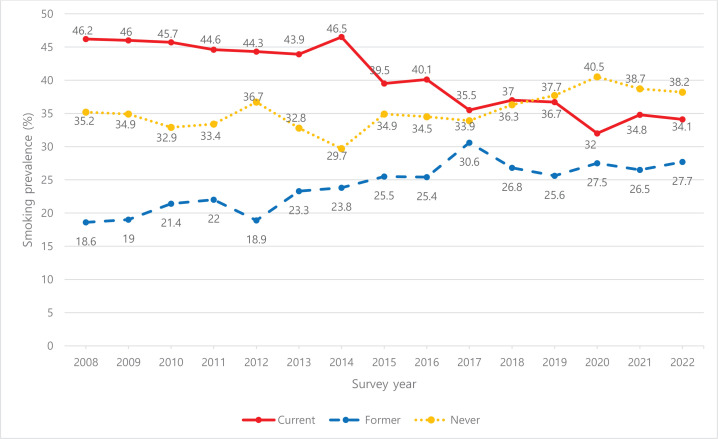
Annual trends in smoking prevalence (%) among career military personnel

Figure 1 illustrates the trend of smoking prevalence among career military personnel from 2008 to 2022. The proportion of ‘current smokers’ decreased from 46.2% in 2008 to 34.1% in 2022. The reduction was gradual from 2008 to 2013, with the most notable decrease to 39.5% occurring in 2015. Despite fluctuations, the overall trend continued downward.

**Figure 2:**
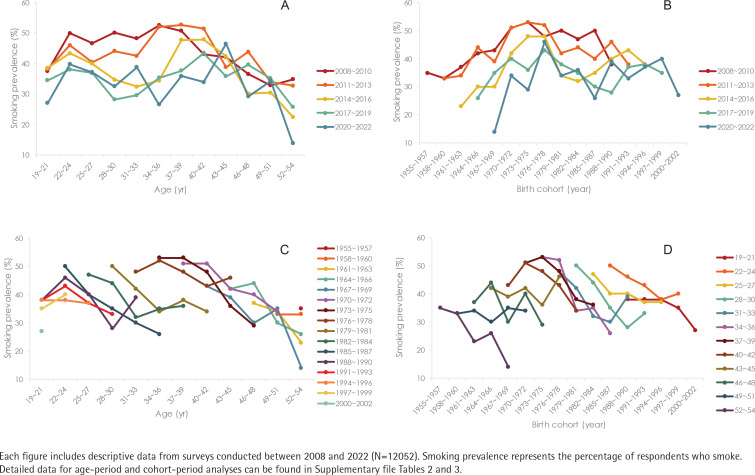
Smoking prevalence (%) among career military personnel by: A) period-age, B) period-birth cohort, C) birth cohort age, and D) age-birth cohort

Figure 2 depicts the variations in smoking prevalence by age, survey year, and birth cohort. For detailed data categorized by age-period (A-P) and cohort-period (C-P), refer to Supplementary file Tables 2 and 3. In Figure 2A, a consistent increase in smoking prevalence for ages 19–21 to 22–24 years was observed across all years. Excluding the coronavirus disease 2019 (COVID-19)-affected years of 2020 to 2022, a marked reduction in smoking prevalence among individuals younger than mid-30s, particularly those aged 34–36 years, occurred between 2014 and 2016. In Figure 2B, recent years exhibited a downward trend in smoking prevalence, with minimal fluctuations in the 1976 to 1978 cohort. Figure 2C shows a significant decrease in smoking prevalence was observed in the 1979 to 1981 cohort, with no consistent pattern across cohorts. In Figure 2D, a marked increase in smoking prevalence from those aged 19–21 to 22–24 years, a peak in late 30s, and a decrease from the mid-30s onwards.

Table 2 shows the age-specific smoking initiation rates among respondents aged ≥34 years who currently smoke, based on data from 2008 to 2019. Only 0.68% of respondents began smoking after the age of 34 years, suggesting that the observed increase in smoking prevalence from mid-30s to early 40s is more likely due to relapse among former smokers rather than new initiations.

**Table 2:** Age-specific smoking initiation (%) among respondents aged 34–54 years who currently smoke by period (N=2247)

	*34–36*	*37–39*	*40–42*	*43–45*	*46–48*	*49–51*	*52–54*
**2008–2010**	0.84 2/238	0.52 1/192	0.89 1/113	1.08 1/93	1.75 1/57	0 0/45	0 0/24
**2011–2013**	0 0/154	1.35 2/148	0 0/125	3.70 2/54	3.51 2/57	2.94 1/34	0 0/17
**2014–2016**	0 0/99	0.87 1/115	1.56 2/128	0 0/71	0 0/34	0 0/31	11.11 1/9
**2017–2019**	0 0/83	0 0/91	0 0/75	0 0/63	0 0/55	0 0/33	0 0/9

Data for smoking initiation age are limited to the years 2008–2019, as information for 2020–2022 was unavailable in KCHS. Each cell shows the percentage and number of participants who began smoking, alongside the total number of participants who currently smoke.

**Figure 3:**
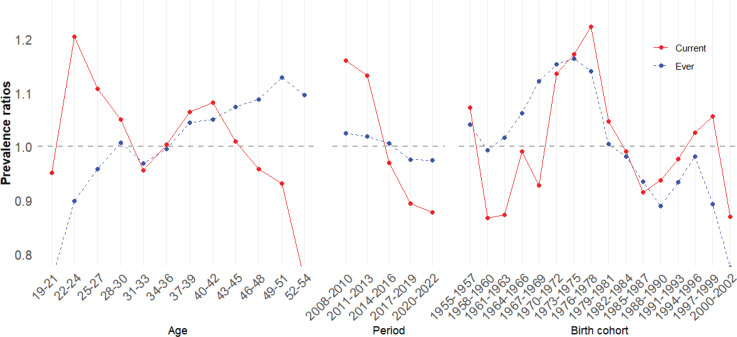
Intrinsic estimator (IE) comparison of smoking prevalence among individuals who currently smoke (current) and who have ever smoked (ever)

Figure 3 illustrates the trends in age, period, and cohort of the IE between ‘current’ and ‘ever’ smokers. The prevalence ratios for ‘current’ and ‘ever’ smoking prevalence are presented in Supplementary file Table 4. ‘Ever’ smokers exhibited a consistent increase in smoking prevalence from the ages of 19–21 up to 30 years; conversely, the smoking prevalence in ‘current’ smokers decreased from the ages of 22–24 years followed by an increase in the early 40s. The smoking prevalence in both groups decreased in early 30s, indicating a reduced rate of smoking initiation at this life stage. Notably, the smoking prevalence in ‘ever’ smokers increased at the age of 37–39 years and continued into the 50s. However, the smoking prevalence in ‘current’ smokers decreased markedly after the age of 43–45 years. The period effect on ‘ever’ smokers mirrored that of ‘current’ smokers but was less pronounced and decreased between 2014 and 2016. The cohort effect demonstrated that individuals born after the 1980s have significantly lower smoking prevalences. Although the 1970s cohort had the highest smoking prevalence, the 1967 to 1969 cohort, despite a higher prevalence ratio of ‘ever’ smokers, showed a marked decrease in ‘current’ smokers.

## DISCUSSION

This study investigated the effects of age, period, and cohort on the smoking prevalence of career military personnel. The smoking prevalence among career military personnel demonstrated a decreasing trend from 46.2% in 2008 to 34.1% in 2022. APC analysis revealed that smoking prevalence increased significantly in early 20s, showed a slight increase in early 40s, and exhibited a downward trend thereafter. The period effect indicated an overall decrease in smoking prevalence from 2008 to 2022, and the cohort effect demonstrated that individuals born in the 1970s had the highest smoking prevalence, whereas those born after the 1980s had a lower smoking prevalence.

In South Korea, career military personnel comprise 34% commissioned officers (including warrant officers) and 66% non-commissioned officers, who typically enlist in their early 20s^25^. The smoking prevalence peaks at the age of 22–24 years due to the stresses of military life, which encourage smoking initiation^4,6,14,26^. Among career military personnel, the proportion of ‘ever’ smokers increased until the age of 50 years, and the decrease in ‘current’ smokers aged 43–45 years suggests enhanced cessation. This may result from rigorous health management or regular physical and fitness examinations in the military^1,27^, or the discharge of military personnel in poor health^7,28^.

The age effect on smoking prevalence observed in this study is consistent with prior research^7,21,24^, except for the increase in smoking prevalence from mid-30s to early 40s. This increase likely results from unique military life stressors, such as economic and occupational instability, which are exacerbated by the rigid structure of military promotion^29^. For example, non-commissioned officers face mandatory retirement if they do not achieve senior status, and officers must retire if they do not reach at least the rank of lieutenant-colonel by their mid-40s, irrespective of their capabilities or desires^30,31^. These pressures, coupled with re-employment challenges and financial concerns typical of this life stage, intensify economic anxiety and job insecurity. Consequently, these factors may contribute to the increase in smoking prevalence among career military personnel in their mid-30s to early 40s, differentiating them from the general population and marking a significant, stress-induced risk factor in this group^32^. Therefore, this trend represents an important risk factor among career military personnel.

The period effect in APC analysis indicates significant shifts in tobacco control policies in the military, particularly between 2006 and 2009 and in 2015. The Plan to Reduce and Eliminate Duty-Free Cigarette Allocation for Soldiers’ Health Promotion, implemented from 2006 to 2009, terminated the provision of duty-free cigarettes but initially targeted conscripted soldiers; thus, it had minimal impact on career military personnel. A 2009 survey revealed that over half of career military personnel reported no change in their smoking habits^27^. The significant budget increase for smoking cessation programs in 2015 expanded eligibility to approximately 358000 military personnel, including many career military personnel previously underserved by such initiatives. This policy change correlated with a noticeable decline in smoking prevalence, particularly among those in their mid-30s after the 2014 to 2016 period. According to Miech et al.^[Bibr CIT0033]33^, policy changes within closed environments like the military can lead to long-term behavior changes. Therefore, the increased budget for tobacco control policies since 2015 could be effective in fostering sustained non-smoking behaviors within the military and promoting lasting behavioral changes even after military service. Individuals born before the 1980s, especially between 1976 and 1978, tended to have a higher prevalence of ‘ever’ smokers, with smoking habits likely formed in the late 1990s during military service. This coincided with the post-1995 National Health Promotion Act, which initiated tobacco control campaigns and public health initiatives^34^. However, these policies, focusing on advertising and activities by public health centers, might have indirectly affected career military personnel due to the military’s insularity^35^, possibly explaining the higher smoking prevalence in this group. Conversely, individuals born in the 1980s grew up under the influence of national tobacco control policies, resulting in lower smoking prevalence and reflecting a societal shift against smoking during their adolescence, a trend corroborated by a prior study^36^.

### Limitations

This study has several limitations. It included only career military personnel who responded to the survey, limiting the generalizability of the results and precluding in-depth analysis of variables such as rank and service duration. The small number of female participants impeded gender-based analysis, which is important for efforts to develop targeted smoking cessation policies. Moreover, the inherent challenges of APC analysis restrict the ability to evaluate the effects of various smoking policies, such as those implemented nationally and within the military.

## CONCLUSIONS

We analyzed 15-year data from KCHS to examine smoking trends among career military personnel. The smoking prevalence decreased from 46.2% in 2008 to 34.1% in 2022. We identified unique smoking patterns, such as an increased prevalence in individuals in their mid-30s to early 40s and a decline between 2014 and 2016, especially among those born in the 1970s. The smoking prevalence progressively decreased in individuals born after the 1980s. The findings underscore the necessity of extending military tobacco control policies, which formerly focused on conscripted soldiers, to career military personnel and highlight the importance of tailoring these policies to enhance health initiatives. Our analysis of smoking trends and the effects of policies on them will inform tobacco control policies and health interventions specifically tailored to career military personnel.

## Supplementary Material



## Data Availability

All data used in this study are accessible following the approval of the Korea Community Health Survey (KCHS) Data Service. Interested researchers must obtain the necessary approval from KCHS to access the data. For more information and to apply for access, please visit the official KCHS website at https://chs.kdca.go.kr/chs/index.do.
