# Supplementary material for: Age-period-cohort analysis of smoking prevalence trends among career military personnel in South Korea
Source: Tob Induc Dis. 2024 Dec 7;22:10.18332/tid/196477. doi: 10.18332/tid/196477 (PMC11624574; doi:10.18332/tid/196477)
Supplement: Supplementary file 1 [file TID-22-185-s1.pdf]

SUPPLEMENTARY FILE

Supplementary file Table 1. Policy timelines in national and military tobacco policies and initiatives

Career military personnel are influenced by the prevalent smoking culture within the military as well as by the national anti-smoking policies. In this table, a timeline format has been utilized to detail both internal military and national policies related to smoking. The focus within the military primarily revolves around smoking cessation support programs. These programs are targeted at conscripted soldiers and conscripted police members. They are conducted in partnership with the Korea Health Promotion Institute, as part of the wider national efforts to curb smoking. Therefore, the section on military internal policies and issues emphasizes policies specialized for the military or issues that have been reported in the media.

Year	National policies and issues	Military policies and issues
**1948**		• Post-establishment of the military, cigarettes uniformly provided as welfare measures
**1967**		• Among those who never smoked given allowances for alternative items such as breath mints, caramels
**1986**	• Enforcement of the Tobacco Business Law<br>- Warning labels on cigarette packages<br>- Restrictions on tobacco advertising	
**1995**	• **Establishment of the national health promotion act**<br>- Designation of no-smoking areas<br>- Prohibition of tobacco sales to adolescents	• **Adoption of a no-smoking policy at recruit training command[1]**<br>- Total ban on smoking during the Basic military training (5-6weeks)
**1998**	• Establishment of the National Health Promotion Fund	• **Establishment of the armed forces health promotion regulation[1]**<br>• Production of no-smoking guides, related booklets, and VTR tapes.
**1999**	• Imposition of a tobacco health promotion surcharge	
**2000**	• Commencement of broadcast of TV non-smoking public service advertisements	
**2002**	• Disclosure of tobacco ingredients (Tar and Nicotine)	
**2003**	• **Signature of WHO FCTC**	• Establishment of smoking

	• Strengthening expansion of no-smoking areas	cessation clinics in the military hospitals[2]
**2004**	• **Start of the smoking cessation clinic pilot project**<br>- At 10 public health centers<br>• **Increase in tobacco prices From 2,000 to 2,500KRW**	
**2005**	• **Ratification of WHO FCTC**<br>• **Start of smoking cessation clinic counseling and treatment service at all public health centers**	• **Complete ban on smoking military hospital and medical units**<br>• Designation of no-smoking areas in military installations
**2006**	• Further expansion of no-smoking areas<br>• **Implementation of the smoking cessation hotline service (Quit-line)**	• **Implementation of the 'Plan to reduce and eliminate duty-free cigarette allocation for soldiers health promotion'[2]**<br>- 2006: Reduction from 15 to 10 packs per month<br>- 2007-2008: Further reduction of 5 packs per month<br>- 2009: Complete termination
**2007**	• Display of carcinogenic substance warning messages<br>• Campaigns to prevent secondhand smoke	• Implementation of the 'Mobile smoking cessation clinic program' through unit visits
**2008**	• 'Say No' campaign	• **Initiation of assigning of division-level nursing officers[3]**<br>- Each unit is responsible for conducting its smoking cessation campaigns and clinics
**2009**	• 'Smoke Free' campaign	• **Complete abolition of duty-free cigarette allocation in the military**<br>• **Launch of smoking cessation support programs for conscripted soldiers and police**
**2010**	• Establishment of legal grounds for imposing fines on smoking within no-smoking zones.	
**2011**	• Strengthening of regulations on warning labels on cigarette packages.	• Pilot programs of smoke-free military units and intensive smoking cessation management units<br>- Smoke-free units: Require complete cessation of smoking over 6months<br>- Intensive smoking cessation management units: Focus on targeted smoking cessation efforts
**2012**	• **Designation of all public facilities as No-smoking zones**<br>• Prohibition of flavoring substances labeling	• Issues Judged by the National Human Rights Commission of Korea[4]<br>- Mandating soldiers in "smoke-free units" to quit smoking is deemed a

		human rights violation.
**2013**	• Complete indoor smoking ban in restaurants over 150㎡<br>• Classification of e-cigarettes as tobacco products<br>• Prohibition of misleading descriptors like 'mild,' 'low tar'<br>• Establishment of regulations for 'non-smoking guidance'	• Transitioned from Designating No-Smoking Areas to Establishing Smoking Areas in the Armed Forces Health Promotion Regulation<br>• Prohibition of tobacco advertising on military bases<br>• Prohibition of tobacco sales at military-medical installations<br>• Strengthening of Air Force non-smoking policies[5]<br>- Designation of all areas as no-smoking zones, later maintaining some smoking areas due to rights infringement concerns<br>- Prohibition of smoking during duty hours for active pilots.
**2014**	• Complete indoor smoking ban in restaurants over 100㎡	
**2015**	**• Major budget boost for National Smoking Cessation Support Program**<br>- Tenfold rise from 11.3 billion KRW in 2014 to 147.5 billion KRW<br>- Partial funding for clinic/hospital smoking cessation treatments<br>**• Increase in tobacco prices from 2,500 to 4,500 KRW**<br>**• Complete indoor smoking ban in all restaurants, coffee shops, regardless of size**	**• Major budget boost for 'Smoking cessation support programs for conscripted soldiers and police'**<br>**- Budget increased from 0.4-0.5 to 5 billion KRW in 2015**<br>**- Target expanded from 8-10% to all who smokes in the military**<br>- Contents: Includes education, training for leaders, campaigns, and clinics
**2016**	• Mandatory implementation of warning images on cigarette packs	• Start of sales of foreign imported cigarettes in PX[7]
**2017**	• Launch of the first domestic heated tobacco product	
**2018**	• Promotion of the harmful effects of Heated tobacco product(HTP)	• Issues Judged by the National Human Rights Commission of Korea[8]<br>- Calls for improvement as mandatory non-smoking policies and punitive measures in the military hospitals are considered human rights violations

**2019**	• Promotion of the harmful effects of new types of tobacco	• Improvement of military culture policies<br>- Allowing the use of smartphones and weekday outings for soldiers after duty hours<br>**• Recommendation to prohibit the use and bring in liquid e-cigarettes within the military base**
**2020-2021**	• Promotion of the 'No Smoking Campaign' for youth smoking prevention	• Consideration of COVID-19 situation: control of soldiers' outings and leaves, application of strict social distancing measures within the military<br>• Offering remote smoking cessation services via phone and mobile apps
**2022**		• Temporary Smoking Allowance in recruit training commands as Cultural Improvement[9,10]<br>- reinstated full ban for smoke-free rights
**- Present**		• Attempts to introduce HTP to PX<br>- Unsuccessful attempts to add HTPs to PX failed Military Welfare Corporation<br>- The military cigarette market is 100 billion KRW, with HTPs projected to reach 20 billion KRW

Abbreviations: HTP, Heated Tobacco Product; PX, Post Exchange; KRW, Korean Won

Supplementary file Table 2. Age-specific smoking prevalence (%) among career military personnel by survey period (2008-2022)

Age/ Year	2008-2010	2011-2013	2014-2016	2017-2019	2020-2022
**Total**	1,745 / 3,821 (45.7%)	1,083 / 2,413 (44.9%)	886 / 2,302 (38.5%)	740 / 2,094 (35.3%)	490 / 1,422 (34.5%)
**19-21**	208 / 554 (37.5%)	85 / 223 (38.1%)	60 / 156 (38.5%)	58 / 168 (34.5%)	24 / 89 (27%)
**22-24**	176 / 353 (49.9%)	80 / 174 (46%)	71 / 164 (43.3%)	65 / 171 (38%)	47 / 118 (39.8%)
**25-27**	167 / 358 (46.6%)	84 / 208 (40.4%)	81 / 203 (39.9%)	79 / 214 (36.9%)	56 / 151 (37.1%)
**28-30**	217 / 433 (50.1%)	112 / 254 (44.1%)	85 / 245 (34.7%)	68 / 241 (28.2%)	53 / 163 (32.5%)
**31-33**	215 / 446 (48.2%)	133 / 313 (42.5%)	102 / 315 (32.4%)	61 / 206 (29.6%)	54 / 139 (38.8%)
**34-36**	238 / 453 (52.5%)	154 / 296 (52%)	99 / 288 (34.4%)	83 / 235 (35.3%)	36 / 136 (26.5%)
**37-39**	192 / 379 (50.7%)	148 / 281 (52.7%)	115 / 241 (47.7%)	91 / 242 (37.6%)	47 / 131 (35.9%)
**40-42**	113 / 262 (43.1%)	125 / 243 (51.4%)	128 / 267 (47.9%)	75 / 173 (43.4%)	43 / 127 (33.9%)
**43-45**	93 / 221 (42.1%)	54 / 139 (38.8%)	71 / 168 (42.3%)	63 / 176 (35.8%)	59 / 127 (46.5%)
**46-48**	57 / 156 (36.5%)	57 / 130 (43.8%)	34 / 113 (30.1%)	55 / 139 (39.6%)	33 / 113 (29.2%)
**49-51**	45 / 137 (32.8%)	34 / 100 (34%)	31 / 102 (30.4%)	33 / 94 (35.1%)	34 / 99 (34.3%)
**52-54**	24 / 69 (34.8%)	17 / 52 (32.7%)	9 / 40 (22.5%)	9 / 35 (25.7%)	4 / 29 (13.8%)

Note: Each cell shows the number of smokers, the total surveyed, and the percentage of smokers.

Supplementary file Table 3. Birth cohort-specific smoking prevalence (%) among career military personnel across survey periods (2008-2022)

Birth cohort / Year	2008-2010	2011-2013	2014-2016	2017-2019	2020-2022
**Total**	1,745 / 3,821 (45.7 %)	1,083 / 2,413 (44.9 %)	886 / 2,302 (38.5 %)	740 / 2,094 (35.3 %)	490 / 1,422 (34.5 %)
**1955-1957**	24 / 69 (34.8 %)				
**1958-1960**	45 / 137 (32.8 %)	17 / 52 (32.7 %)			
**1961-1963**	57 / 156 (36.5 %)	34 / 100 (34 %)	9 / 40 (22.5 %)		
**1964-1966**	93 / 221 (42.1 %)	57 / 130 (43.8 %)	31 / 102 (30.4 %)	9 / 35 (25.7 %)	
**1967-1969**	113 / 262 (43.1 %)	54 / 139 (38.8 %)	34 / 113 (30.1 %)	33 / 94 (35.1 %)	4 / 29 (13.8 %)
**1970-1972**	192 / 379 (50.7 %)	125 / 243 (51.4 %)	71 / 168 (42.3 %)	55 / 139 (39.6 %)	34 / 99 (34.3 %)
**1973-1975**	238 / 453 (52.5 %)	148 / 281 (52.7 %)	128 / 267 (47.9 %)	63 / 176 (35.8 %)	33 / 113 (29.2 %)
**1976-1978**	215 / 446 (48.2 %)	154 / 296 (52 %)	115 / 241 (47.7 %)	75 / 173 (43.4 %)	59 / 127 (46.5 %)
**1979-1981**	217 / 433 (50.1 %)	133 / 313 (42.5 %)	99 / 288 (34.4 %)	91 / 242 (37.6 %)	43 / 127 (33.9 %)
**1982-1984**	167 / 358 (46.6 %)	112 / 254 (44.1 %)	102 / 315 (32.4 %)	83 / 235 (35.3 %)	47 / 131 (35.9 %)
**1985-1987**	176 / 353 (49.9 %)	84 / 208 (40.4 %)	85 / 245 (34.7 %)	61 / 206 (29.6 %)	36 / 136 (26.5 %)
**1988-1990**	208 / 554 (37.5 %)	80 / 174 (46 %)	81 / 203 (39.9 %)	68 / 241 (28.2 %)	54 / 139 (38.8 %)
**1991-1993**		85 / 223 (38.1 %)	71 / 164 (43.3 %)	79 / 214 (36.9 %)	53 / 163 (32.5 %)
**1994-1996**			60 / 156 (38.5 %)	65 / 171 (38 %)	56 / 151 (37.1 %)
**1997-1999**				58 / 168 (34.5 %)	47 / 118 (39.8 %)
**2000-2002**					24 / 89 (27 %)

Note: Each cell shows the number of smokers, the total surveyed, and the percentage of smokers.

Supplementary file Table 4. Intrinsic Estimator - Estimated prevalence ratios of age, period, and birth cohort effects on smoking prevalence among respondents who currently smoke

	Prevalence ratio	Standard error	z-score	P-value	95% Confidence Interval (CI)
Intercept	2.691	0.021	46.740	0.000	(2.650, 2.732)
Age					
**19-21**	0.951	0.047	-1.080	0.282	(0.863, 1.048)
**22-24**	1.204	0.043	4.350	0.000	(1.123, 1.291)
**25-27**	1.108	0.041	2.500	0.013	(1.030, 1.191)
**28-30**	1.051	0.039	1.260	0.207	(0.977, 1.130)
**31-33**	0.967	0.040	-1.100	0.270	(0.892, 1.049)
**34-36**	1.003	0.041	0.090	0.929	(0.926, 1.087)
**37-39**	1.065	0.042	1.500	0.132	(0.986, 1.151)
**40-42**	1.082	0.046	1.730	0.084	(0.995, 1.176)
**43-45**	1.010	0.051	0.200	0.845	(0.915, 1.115)
**46-48**	0.958	0.058	-0.740	0.460	(0.851, 1.079)
**49-51**	0.931	0.064	-1.110	0.268	(0.814, 1.065)
**52-54**	0.752	0.115	-2.470	0.013	(0.557, 1.015)
**Period**					
**2008-2010**	1.161	0.021	6.940	0.000	(1.121, 1.203)
**2011-2013**	1.133	0.024	5.120	0.000	(1.087, 1.181)
**2014-2016**	0.970	0.026	-1.190	0.233	(0.920, 1.022)
**2017-2019**	0.893	0.027	-4.090	0.000	(0.842, 0.948)
**2020-2022**	0.878	0.034	-3.840	0.000	(0.814, 0.947)

Supplementary file Table 4. (continued)

	Prevalence ratio	Standard error	z-score	P-value	95% Confidence Interval (CI)
Birth cohort					
**1955-1957**	1.072	0.176	0.400	0.690	(0.760, 1.516)
**1958-1960**	0.868	0.104	-1.370	0.171	(0.708, 1.063)
**1961-1963**	0.874	0.086	-1.580	0.115	(0.739, 1.034)
**1964-1966**	0.992	0.069	-0.120	0.904	(0.866, 1.136)
**1967-1969**	0.928	0.064	-1.170	0.242	(0.820, 1.051)
**1970-1972**	1.135	0.053	2.380	0.017	(1.022, 1.261)
**1973-1975**	1.172	0.050	3.170	0.002	(1.063, 1.293)
**1976-1978**	1.223	0.048	4.170	0.000	(1.113, 1.345)
**1979-1981**	1.047	0.046	1.000	0.318	(0.956, 1.147)
**1982-1984**	0.991	0.045	-0.200	0.842	(0.906, 1.083)
**1985-1987**	0.915	0.044	-1.990	0.046	(0.839, 0.999)
**1988-1990**	0.937	0.040	-1.600	0.110	(0.866, 1.015)
**1991-1993**	0.976	0.049	-0.480	0.629	(0.887, 1.074)
**1994-1996**	1.026	0.060	0.440	0.661	(0.913, 1.154)
**1997-1999**	1.057	0.075	0.730	0.464	(0.911, 1.225)
**2000-2002**	0.870	0.168	-0.830	0.407	(0.626, 1.209)

Supplementary file Table 4-1. Intrinsic Estimator - Estimated prevalence ratios of age, period, and birth cohort effects on smoking prevalence among respondents who previously smoke

	Prevalence ratio	Standard error	z-score	P-value	95% Confidence Interval (CI)
Intercept	0.632	0.0119	-38.580	0.000	(0.617, 0.647)
Age					
**19-21**	0.752	0.0286	-9.930	0.000	(0.711, 0.796)
**22-24**	0.899	0.0268	-3.960	0.000	(0.853, 0.948)
**25-27**	0.959	0.0245	-1.720	0.085	(0.914, 1.006)
**28-30**	1.007	0.0228	0.320	0.752	(0.963, 1.053)
**31-33**	0.969	0.0228	-1.380	0.166	(0.927, 1.013)
**34-36**	0.996	0.0227	-0.170	0.863	(0.953, 1.041)
**37-39**	1.044	0.023	1.890	0.058	(0.998, 1.093)
**40-42**	1.051	0.0243	2.030	0.042	(1.002, 1.102)
**43-45**	1.074	0.0263	2.700	0.007	(1.020, 1.130)
**46-48**	1.088	0.0287	2.930	0.003	(1.028, 1.151)
**49-51**	1.128	0.0308	3.930	0.000	(1.062, 1.199)
**52-54**	1.097	0.0503	1.830	0.067	(0.994, 1.210)
**Period**					
**2008-2010**	1.025	0.012	2.020	0.043	(1.001, 1.049)
**2011-2013**	1.02	0.014	1.390	0.164	(0.992, 1.048)
**2014-2016**	1.006	0.0142	0.440	0.663	(0.979, 1.035)
**2017-2019**	0.976	0.015	-1.640	0.101	(0.947, 1.005)

**2020-2022**	0.975	0.0181	-1.400	0.160	(0.941, 1.010)

Supplementary file Table 4-1. (continued)

	Prevalence ratio	Standard error	z-score	P-value	95% Confidence Interval (CI)
Birth cohort					
**1955-1957**	1.041	0.0838	0.480	0.633	(0.883, 1.227)
**1958-1960**	0.994	0.0508	-0.120	0.901	(0.899, 1.098)
**1961-1963**	1.017	0.0422	0.390	0.697	(0.936, 1.104)
**1964-1966**	1.062	0.0352	1.720	0.085	(0.992, 1.138)
**1967-1969**	1.122	0.0316	3.650	0.000	(1.054, 1.194)
**1970-1972**	1.154	0.0279	5.120	0.000	(1.092, 1.219)
**1973-1975**	1.164	0.0269	5.660	0.000	(1.105, 1.227)
**1976-1978**	1.14	0.0268	4.900	0.000	(1.082, 1.202)
**1979-1981**	1.005	0.0263	0.190	0.846	(0.954, 1.058)
**1982-1984**	0.982	0.0262	-0.710	0.479	(0.932, 1.033)
**1985-1987**	0.935	0.0265	-2.550	0.011	(0.887, 0.985)
**1988-1990**	0.89	0.0251	-4.650	0.000	(0.847, 0.935)
**1991-1993**	0.933	0.0301	-2.290	0.022	(0.880, 0.990)
**1994-1996**	0.982	0.0363	-0.500	0.615	(0.915, 1.054)
**1997-1999**	0.893	0.0483	-2.340	0.019	(0.812, 0.982)
**2000-2002**	0.776	0.1047	-2.430	0.015	(0.632, 0.952)

Supplementary References

1. Jeong SJ, Lee CG, Lyu SY, Kim GS. The Smoking Status and Its Associated Factors of Some Army Soldiers. Tuberculosis and Respiratory Diseases. 2001;51(5):426-436. doi:10.4046/trd.2001.51.5.426
2. Cho k-s. FCTC and tobacco control policy in Korea. Health Welfare Policy Forum. 2006;116:7-23.
3. Hyun J-s, Na J-s, Yang J-Y, Oh Y-r. Survey of NurseOfficers' Works in Divisional Health care Unit. Journal of military nursing research. 2013;31(1):56-75.
4. Non-smoking military units should be operated through the voluntary participation of soldiers (금연부대 운영, 장병의 자발적 참여로 이루어져야). National Human Rights Commission of Korea; Mach 29, 2012, Accessed May 3, 2024. https://www.humanrights.go.kr/site/program/board/basicboard/view?boardtypeid=24&boardid=604396
5. Air Force to Launch 'War Against Tobacco' Starting Next Month (공군, 다음 달 부터 '담배와의 전쟁'). The Korea Defense Daily. Jun 28, 2013. Accessed May 3, 2024. https://www.korea.kr/news/policyNewsView.do?newsId=148763301&call_from=naver_news#goList
6. Yu G-S. Smoking and Air Quality - Framework Convention on Tobacco Control (FCTC), National Smoking Cessation Policies, and Future Challenges. *Air Cleaning Technology*. 2015;28(2):1-10.
7. Lim H-j. Foreign Cigarettes Sold at PX (PX서 판매되는 외산담배). Yonhap News. May 25, 2016. Accessed March 25, 2024. https://www.yna.co.kr/view/PYH20160525045700013?input=1196m
8. Maeng S-y. Constitutional Court Rules in Favor of 'Privacy Rights': Smoking Rights Also Guaranteed as Fundamental Rights (헌재 ‘혐연권 우위’ 판결…흡연권도 기본권으로 보장). The Korea Defense Daily. November 26, 2018. Accessed May 3, 2024. https://kookbang.dema.mil.kr/newsWeb/20181127/1/BBSMSTR_000000100061/view.do
9. Baek S-h. Controversy Over Basic Rights: What is the Final Decision on Smoking by Recruit Soldiers in the Military? (기본권 문제라는데…훈련병 흡연, 軍 최종 결정

은?). KBS News. February 10, 2022. Accessed May 3, 2024. https://news.kbs.co.kr/news/pc/view/view.do?ncd=5391451

10. Lee S-m. Army recruit training command Reinstates 'Total Smoking Ban'... 'Issues such as lack of facilities identified' (육군훈련소 다시 ‘전면 금연’…'시설 미구비 등 문제점 식별'). KBS News. February 8, 2023. Accessed May 3, 2024. https://news.kbs.co.kr/news/pc/view/view.do?ncd=7600240

© 2024 Jung S. et al.
